# Molecular Pathways of Vulnerable Carotid Plaques at Risk of Ischemic Stroke: A Narrative Review

**DOI:** 10.3390/ijms25084351

**Published:** 2024-04-15

**Authors:** Giuseppe Miceli, Maria Grazia Basso, Chiara Pintus, Andrea Roberta Pennacchio, Elena Cocciola, Mariagiovanna Cuffaro, Martina Profita, Giuliana Rizzo, Antonino Tuttolomondo

**Affiliations:** 1 Department of Health Promotion, Mother and Child Care, Internal Medicine and Medical Specialties (ProMISE), University of Palermo, Piazza delle Cliniche 2, 90127 Palermo, Italy; miceli.gpp@gmail.com (G.M.); mariagbasso.92@gmail.com (M.G.B.); chiarapintus1809@gmail.com (C.P.); pennacchio.andrea@libero.it (A.R.P.); elena.cocciola@gmail.com (E.C.); marinecuffaro@libero.it (M.C.); martinaprofita9@gmail.com (M.P.); giulianarizzo992@gmail.com (G.R.); 2 Internal Medicine and Stroke Care Ward, University Hospital, Policlinico “P. Giaccone”, 90127 Palermo, Italy

**Keywords:** carotid plaque, stroke, vulnerability, inflammation

## Abstract

The concept of vulnerable carotid plaques is pivotal in understanding the pathophysiology of ischemic stroke secondary to large-artery atherosclerosis. In macroscopic evaluation, vulnerable plaques are characterized by one or more of the following features: microcalcification; neovascularization; lipid-rich necrotic cores (LRNCs); intraplaque hemorrhage (IPH); thin fibrous caps; plaque surface ulceration; huge dimensions, suggesting stenosis; and plaque rupture. Recognizing these macroscopic characteristics is crucial for estimating the risk of cerebrovascular events, also in the case of non-significant (less than 50%) stenosis. Inflammatory biomarkers, such as cytokines and adhesion molecules, lipid-related markers like oxidized low-density lipoprotein (LDL), and proteolytic enzymes capable of degrading extracellular matrix components are among the key molecules that are scrutinized for their associative roles in plaque instability. Through their quantification and evaluation, these biomarkers reveal intricate molecular cross-talk governing plaque inflammation, rupture potential, and thrombogenicity. The current evidence demonstrates that plaque vulnerability phenotypes are multiple and heterogeneous and are associated with many highly complex molecular pathways that determine the activation of an immune-mediated cascade that culminates in thromboinflammation. This narrative review provides a comprehensive analysis of the current knowledge on molecular biomarkers expressed by symptomatic carotid plaques. It explores the association of these biomarkers with the structural and compositional attributes that characterize vulnerable plaques.

## 1. Introduction

Stroke represents one of the leading causes of death and disability worldwide, with carotid artery atherosclerosis being a primary risk factor for its development [1]. The concept of vulnerable carotid plaques is pivotal in understanding the pathophysiology of ischemic stroke secondary to large-artery atherosclerosis (LAAS), one of the most frequent causes according to the classification system of the trial of ORG 10,172 in the acute stroke treatment (TOAST). Vulnerable plaques are prone to rupture, which can precipitate a cascade leading to thromboembolic episodes and ischemic strokes [2]. Vulnerable plaques are responsible for 0.5–1% of ischemic strokes every year [3]. The occurrence of vulnerable plaques, as determined by different imaging criteria, comprises approximately 25% of all asymptomatic carotid plaques [4]. Plaques identified as vulnerable are linked to an increased likelihood of experiencing an ipsilateral ischemic event. This correlation is similarly evident among plaques with less than 50% luminal narrowing in patients with cryptogenic stroke, particularly those with embolic stroke of an undetermined source [5].

Moreover, symptomatic carotid plaques, the ones associated with clinical symptoms such as transient ischemic attacks, particularly underscore the imminent danger of such events. The biological complexity of carotid plaque vulnerability necessitates a multifaceted approach to its analysis. Traditionally, these plaques have been investigated through their anatomical and morphological characteristics [6]. In macroscopic evaluation, vulnerable plaques are characterized by one or more of the following features: microcalcification; neovascularization; lipid-rich necrotic cores (LRNCs); intraplaque hemorrhage (IPH); thin fibrous caps; plaque surface ulceration; huge dimensions, suggesting stenosis; and plaque rupture. Recognizing these macroscopic characteristics is crucial for estimating the risk of cerebrovascular events, also in the case of non-significant (less than 50%) stenosis [7,8,9].

Delineating these plaques’ hallmarks is critical for developing strategies to preempt ischemic stroke and mitigate its hazardous consequences [10]. However, the advent of new technologies together with artificial intelligence, and the evolution of biochemical insights have shifted the focus towards understanding and identifying molecular patterns that denote plaque instability [11,12,13]. Inflammatory biomarkers, such as cytokines and adhesion molecules, lipid-related markers like oxidized low-density lipoprotein, and proteolytic enzymes capable of degrading extracellular matrix components are among the key molecules that are scrutinized for their associative roles in plaque instability. Through their quantification and evaluation, these biomarkers reveal intricate molecular cross-talk governing plaque inflammation, rupture potential, and thrombogenicity [14]. They offer a lens through which the nuances of plaque pathophysiology can be discerned, aiding in the stratification of stroke risk and the personalization of therapeutic interventions. This narrative review provides a comprehensive analysis of the current knowledge on molecular biomarkers expressed by symptomatic carotid plaques. It explores the association of these biomarkers with the structural and compositional attributes that characterize vulnerable plaques, and their potential role as predictors of clinical outcomes, providing insights into potential future directions in the therapeutic targeting of molecular biomarkers.

## 2. Vulnerable Plaques in the Proteomic Era

Recent advancements in proteomics have revolutionized our understanding of the molecular intricacies underlying vulnerable carotid plaques, offering unprecedented insights into the pathogenesis of atherosclerosis and its clinical implications [11]. Carotid plaque vulnerability, characterized by the propensity for rupture and subsequent thromboembolic events, represents a critical determinant of stroke risk [3]. Traditional diagnostic modalities, such as imaging techniques and biomarker assays, have provided valuable insights into plaque morphology and composition. However, the identification of specific molecular signatures associated with plaque vulnerability remained a daunting challenge until the advent of proteomic technologies [12].

Proteomics, the large-scale study of proteins and their functions, has emerged as a powerful tool for dissecting the complex proteomic landscape of carotid plaques. High-throughput mass spectrometry-based proteomic approaches enable the simultaneous identification and quantification of thousands of proteins within a single sample, providing comprehensive insights into the molecular alterations underlying plaque instability [14].

Recent proteomic studies have revealed a diverse array of protein alterations associated with vulnerable carotid plaques, encompassing multiple biological processes implicated in plaque pathophysiology. One of the key findings is the dysregulation of proteins involved in extracellular matrix (ECM) remodeling, such as matrix metalloproteinases (MMPs) and their inhibitors, tissue inhibitors of metalloproteinases (TIMPs). MMPs are a family of proteolytic enzymes capable of degrading various components of the ECM, including collagen, elastin, and proteoglycans [14]. Excessive MMP activity has been implicated in plaque destabilization by promoting fibrous cap thinning and collagen degradation, rendering the plaque more prone to rupture. Conversely, the upregulation of TIMPs serves as a counter-regulatory mechanism to inhibit MMP activity and maintain ECM integrity, thus contributing to plaque stabilization [8].

In addition to ECM remodeling, proteomic studies have identified alterations in proteins involved in inflammation, oxidative stress, lipid metabolism, and thrombosis within vulnerable carotid plaques. Inflammatory mediators, such as cytokines, chemokines, and adhesion molecules, play a crucial role in orchestrating the inflammatory response within the plaque microenvironment, contributing to endothelial dysfunction, leukocyte recruitment, and foam cell formation. Oxidative stress, characterized by an imbalance between reactive oxygen species (ROS) production and antioxidant defense mechanisms, promotes lipid peroxidation, endothelial dysfunction, and vascular inflammation, thereby exacerbating plaque instability. The dysregulation of lipid metabolism-related proteins, such as apolipoproteins and lipid transporters, further contributes to lipid accumulation and foam cell formation within the plaque. Moreover, alterations in proteins involved in thrombosis, including coagulation factors, platelet receptors, and fibrinolytic enzymes, predispose vulnerable plaques to thrombotic events and subsequent ischemic stroke.

The identification of specific protein biomarkers associated with vulnerable carotid plaques holds immense clinical promise for risk stratification, diagnostic imaging, and targeted therapeutic interventions [9]. The proteomic profiling of plaque specimens obtained via carotid endarterectomy or minimally invasive imaging-guided biopsy techniques allows for the identification of candidate biomarkers indicative of plaque vulnerability. These biomarkers can serve as diagnostic indicators for identifying patients at high risk of stroke and guide personalized treatment strategies aimed at stabilizing vulnerable plaques and preventing adverse cardiovascular events [8].

## 3. Risk Factors and Pathophysiology of the Vulnerability of Carotid Plaques

Carotid plaque instability, a critical factor in the pathogenesis of ischemic stroke, is influenced by various cardiovascular risk factors. Hypertension stands as a prominent contributor, exerting mechanical stress on the vessel wall and fostering plaque vulnerability. Dyslipidemia, characterized by elevated LDL cholesterol and reduced HDL cholesterol levels, promotes atherosclerosis progression and plaque destabilization. In addition, diabetes mellitus induces endothelial dysfunction and oxidative stress, accelerating plaque formation and inflammation. Smoking exacerbates plaque instability through endothelial damage and a heightened inflammatory response. Furthermore, aging and male gender are generally associated with increased plaque vulnerability. Interestingly, Montanaro et al. [15] demonstrated that plaque instability is associated with the high in situ expression of some cytokines, such as IL-2, IL-6, and IL-17, but surprisingly none of the classic cardiovascular risk factors analyzed showed a significant association between the in situ expression of the abovementioned markers and plaque instability. Moreover, a significant increase in IL-6-positive and IL-17-positive cells was encountered in unstable atheromatous plaques of female patients, as compared with unstable plaques of male patients.

The risk stratification for cerebrovascular events is typically based on the identification of symptomatic plaques in patients with carotid atheromas. However, asymptomatic plaques may be responsible for acute cerebrovascular events after their ulceration, rupture, thrombosis, and distal embolization as a consequence of the vulnerability of the lesions. These macroscopic modifications are the result of a complex molecular and endothelial interaction that involves the activation of inflammatory and thrombogenic pathways, leading to plaque instability and a high risk of stroke. For this reason, researchers have recently pointed out the importance of discerning the molecular mechanisms accountable for forming vulnerable plaques. Histologically, vulnerable plaques are characterized by a high amount of lipids, macrophages, and *T lymphocytes* and a lower accumulation of vascular smooth muscle cells (VSMCs) and extracellular matrix compounds [16,17]. The main mechanisms involved in the vulnerability of the carotid plaques are inflammation, the degradation of the ECM, and lipid metabolism.

### 3.1. Role of Inflammation in Plaque Vulnerability

Stable and unstable plaques show different cellular compositions and express different proteins on their surface, suggesting the involvement of different inflammatory pathways in determining the instability of atherosclerotic plaques. Several immune cells participate in atherosclerotic disease, such as macrophages, dendritic cells, monocytes, and T cell lymphocytes. *Macrophages* are the primary cells involved in the development of atherosclerotic lesions. Different signals from the surrounding microenvironment can induce the differentiation of these cells into different phenotypes responsible for the vulnerability of the plaques [18]. The main macrophage subtypes are M1, M2, M (Hb), Mhem, Mox, and M4. M1 macrophages have a proinflammatory phenotype, secreting some cytokines such as IL-1, IL-6, and tumor necrosis factor (TNF)-α and inducing tissue damage through ROS production [19]. Moreover, M2 macrophages express on their surface some chemokine receptors (CXCL-9, CXCL-10, and CXCL-5), promoting the recruitment of circulating cells into the vascular layer [20]. These mechanisms are responsible for the progression of the atherosclerotic plaques.

On the contrary, M2 macrophages inhibit inflammation. This role is achieved through the production of anti-inflammatory cytokines such as IL-10 and TNF-b [21] and profibrotic proteins such as fibronectin, insulin-like growth factor, and transforming growth factor (TGF)-b, which are responsible for tissue repair [22].

M (Hb) and Mhem macrophages result from the accumulation of hemoglobin in resident macrophages after IPH. The expression of transporters responsible for cholesterol efflux (LXRa, LXRb, and ABC transporters ABCA1 and ABCG1) prevents the progression of atherosclerosis and stabilizes the plaques [23,24].

The expression of antioxidant enzymes in the Mox macrophages, derived from the accumulation of oxidized phospholipids, provides an anti-inflammatory role to these cells [25], while a proinflammatory phenotype is attributed to M4 macrophages [26].

Thus, macrophage polarization into the M1 or M2 phenotype determines the vulnerability of the plaque. In the early stages of the atherosclerotic disease, M2 macrophages are the main cells in the lesions. However, with the progression of the disease, M1 cells become predominant, increasing the risk of rupture and cerebral complications [27,28]. Moreover, M1 macrophages are predominantly in the shoulder and the necrotic core of the plaque, while M2 macrophages infiltrate nearby newly formed blood vessels [29].

**Figure 1:**
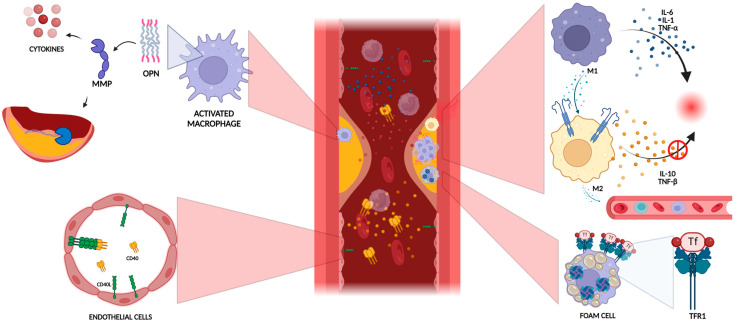
The role of macrophage activation and polarization in plaque vulnerability. The presence of antioxidant enzymes in Mox macrophages, stemming from the accumulation of oxidized phospholipids, confers them an anti-inflammatory function. The polarization of macrophages into M1 or M2 phenotypes plays a crucial role in determining plaque vulnerability. Initially, M2 macrophages predominate in lesions during the early stages of atherosclerotic disease. However, as the disease progresses, there is a shift towards a predominance of M1 cells, heightening the risk of plaque rupture and cerebral complications. Furthermore, M1 macrophages are primarily located in the plaque’s shoulder and necrotic core, while M2 macrophages tend to infiltrate areas near newly formed blood vessels. Macrophages exhibit plasticity in their polarization in response to environmental stimuli. Polarization can shift phenotypes from M1 to M2 in response to IL-4, and vice versa following induction by lipopolysaccharide and IFN-g. The balance between M1 and M2 phenotypes is a dynamic determinant of atherosclerotic plaque vulnerability and the likelihood of acute cerebrovascular events (Created with BioRender.com).

Macrophages can change their polarization depending on stimuli from the surrounding environment. In his study, Khallou-Laschet demonstrated the induction of polarization from M1 to M2 phenotypes by IL-4 and vice versa after lipopolysaccharide and IFN-g induction [30]. The M1/M2 ratio is a mutable condition determining the vulnerability of the atherosclerotic plaques and the risk of acute cerebrovascular events (Figure 1).

Another mechanism through which macrophages induce plaque vulnerability is ferroptosis. Iron accumulation in atherosclerotic lesions is much higher than in healthy vascular walls [31]. The iron overload in macrophages induces ferroptosis, a mechanism of cell death induced by lipid peroxidation, leading to mitochondrial dysfunction and ROS production [32]. Foam cells overexpress transferrin receptor 1, a cell surface receptor involved in transferrin-mediated iron uptake and ferritin synthesis, determining higher iron accumulation in macrophages. Ferroptosis induces macrophage death and increases the dimension of the necrotic core, favoring plaque vulnerability.

Moreover, in the late stages of differentiation, macrophages release the protein YKL-40, which is responsible for different processes such as cell migration, angiogenesis, the regulation of hyaluronic acid synthesis, matrix metalloprotease (MMP)-9 activity, and tissue remodeling. Higher serum levels have recently been related to high plaque vulnerability [33].

Another molecule produced by macrophages and involved in plaque stability is osteopontin (OPN). OPN is produced by activated macrophages from several stimuli, such as cytokines, ILs, and TGFβ [34]. Once released, OPN promotes the recruitment of monocytes and their adhesion to the endothelium, induces the expression of MMPs (in particular MMP-9), favors the degradation of the ECM, and stimulates cytokine expression from macrophages [35]. Moreover, elevated concentrations of OPN have been detected in ulcerated, hemorrhagic, necrotic, and inflamed plaques, suggesting that this protein might be used as a biomarker of plaque instability [36]. So, OPN has a crucial role in the progression of atherosclerotic plaques.

One more signaling molecule involved in plaque vulnerability is derived from the binding of CD40 and CD40L. CD40 is expressed in endothelial cells, while CD40L is present in two forms (cell membrane-bound and soluble) and is released from CD4+ T cells, platelets, monocytes, macrophages, B cells, and natural killer cells [37]. After CD40–CD40L binding, endothelial cells express adhesion factors (selectin-E, vascular cell adhesion molecule, and intercellular adhesion molecule-1), recruit proinflammatory cells through the release of chemotactic molecules (IL-8, RANTES—regulated upon activation, normal T cell expressed and secreted), and release proinflammatory cytokines and procoagulant tissue factor (TF) [38]. Activated platelets expose CD40L on the surface and release the molecule’s soluble form after binding the endothelium. Elevated concentrations of sCD40L have been found in the serum of patients with acute cerebrovascular ischemia compared with controls. In particular, higher levels have been found in the case of LAAS stroke but not in the case of carotid artery stenosis without cerebrovascular disease, underlying the pathogenic role of this pathway in determining plaque progression and instability [39].

On the other hand, VSMCs are responsible for plaque stabilization. Inflammation-induced VSMC activation leads to extracellular matrix and collagen secretion, reinforcing the fibrotic cap. At the same time, the loss of VSCMs is related to the enlargement of the necrotic core and plaque instability. VSCMs produce CTH-H2S (cystathionine gamma-lyase-hydrogen sulfide), which has a protective role in atherosclerosis and whose levels are decreased in atheromas in comparison with the healthy vascular wall [40]. VSCMs accumulating ox-LDL lack CTH-H2S and undergo a mechanism of defective autophagy inducing cell death and increasing the vulnerability of atherosclerotic plaques [41].

Considering the ability of immune cells to produce proinflammatory molecules, atherosclerotic lesions present different protein expressions on their surface based on the plaque vulnerability. Some of these proteins can be detected in the serum and used as circulating biomarkers indicating the risk of cerebrovascular events.

Compared with stable plaques, vulnerable lesions show a higher expression of CRP, high-sensitivity C-reactive protein (hs-CRP), and TNF-α [42,43], and, in particular, the lesions responsible for acute events express a higher concentration of IL-6, IL-17A, IL-18, IL 21, and IL-23 [44,45] and exhibit lower expression of INF-γ [16] in comparison with asymptomatic plaques.

Other proinflammatory proteins detected in higher concentrations in the serum of symptomatic patients compared to asymptomatic patients are soluble urokinase-type plasminogen activator receptors (suPARs), which correlate with plaque inflammation [46], and S100A12, a ^Ca2+^-binding protein belonging to the calgranulin family, whose concentration depends on INF-γ and IL-1 expression [47].

Low circulating levels of Fractalkine and an increased serum concentration of platelet-derived growth factor B (PDGF-BB) are positively related to plaque calcification and the LNRC and represent circulating biomarkers of plaque instability [48]. Fractalkine and PDGF-BB recruit macrophages to the vascular wall, induce intraplaque neoangiogenesis, and induce the switch of VSMCs into the osteogenic phenotype, favoring plaque instability [49,50].

### 3.2. Extracellular Matrix Degradation

MMPs play a critical role in determining the instability of plaques because of their capability to degrade the cellular matrix, the vascular wall, and fibrotic caps, leading to a higher risk of rupture. Different MMP isoforms exist, but not all contribute similarly to atherosclerotic disease.

A higher expression of MMP-9 has been detected on the serum and the surface of unstable plaques [51]. MMP-9 degrades collagen V, promoting the damage of the fibrotic cap, exposing the subendothelial matrix, and attracting other proinflammatory circulating cells. As a confirmation of the role of MMP-9 in plaque vulnerability, Wang et al. have proved that the inhibition of MMP-9 is related to a reduction in plaque instability [52].

Studies carried out on histopathological slices from carotid endarterectomies in patients with acute cerebrovascular events have shown increased concentrations of MMP-9, with statistically significant differences in cases of microembolization or histologic characteristics of plaque instability [53], suggesting a pathogenetic role of this molecule in determining the vulnerability of atherosclerotic lesions, with possible novel future therapeutic targets.

Moreover, vulnerable plaques express higher levels of MMP-1, MMP-2, MMP-7, MMP-8, MMP-12, and MMP-14 and lower concentrations of tissue inhibitor of metalloproteinases 3 [54].

A Disintegrin and metalloproteinase with Thrombospondin motifs (ADAMTS)-4 is another molecule involved in many pathways, such as inflammation, angiogenesis, coagulation, and organ development. This protein is released from macrophages, arterial VSMCs, and endothelial cells, and its expression is induced by proinflammatory cytokines such as IL-1, IL-6, TNF-α, and leptin [55,56].

ADAMTS4 has been found to be highly expressed in the shoulder, adjacent lipid core region, macrophage-rich regions, and fibrous caps of carotid plaques, where this protein can degrade versican, which is responsible for plaque stability and whose levels are decreased in vulnerable lesions [57,58].

### 3.3. Lipid Metabolism

The prevalence of lipidic compounds in the fibrotic component is one of the main determinants of the vulnerability of the plaques.

Lectin-like oxidized low-density lipoprotein receptor-1 is a scavenger receptor expressed on the arterial wall that can bind LDLs, inducing endothelial dysfunction and the progression of atherosclerosis [59]. The extracellular domain of l-1 is released in the soluble form, so increased serum levels are a potential circulating biomarker of plaque instability [60].

Non-high-density lipoprotein cholesterol particles can activate inflammatory and endothelial cells and can contribute to the complication of atheromas. Serum non-high-density lipoprotein cholesterol is considered an independent risk factor for plaque vulnerability [61,62].

One of the significant features of plaque vulnerability is the intraplaque presence of cholesterol crystals. Lesions containing cholesterol crystals have a proinflammatory phenotype because of the activation of the NOD-like receptor family pyrin domain containing 3 (NLRP3) inflammasome and complement system pathways, leading to the expression of inflammatory cytokines such as IL-1, IL-1β, and TNFα. The local expression of these proteins induces the accumulation of macrophages and calcifications, responsible for more vulnerability [63,64].

Moreover, high levels of free fatty acids are related to a higher risk of cerebrovascular events due to the accumulation of micelles and fatty acid vesicles driving plaque instability [65].

## 4. Plaque Rupture and Atherothrombosis

As atherosclerotic disease progresses, plaques are at a greater risk of developing complications. The main complication of the atherosclerotic vulnerable lesions is rupture and, subsequently, atherothrombosis.

Plaque rupture depends not only on the morphologic characteristics of the lesion but also on extrinsic factors. Thus, local dynamic forces, in particular, low shear stress, induce the activation of endothelial cells and leucocytes, upregulating proinflammatory processes, which provoke the increased vulnerability of the lesions [66]. Moreover, circumference forces acting on the fibrotic cap shoulder, where there is the primary concentration of proinflammatory cells, contribute to plaque rupture [67].

According to Slager et al. [68,69], in the presence of risk factors for atherosclerosis, low shear stress contributes to endothelial dysfunction in local areas and the accumulation of eccentric plaques, while normal-to-high shear stress is protective against atherosclerosis. As these plaques grow, those located at preserved lumen sites experience heightened tensile stress at their edges, increasing their susceptibility to fissuring and thrombosis. Subsequently, the loss of the plaque-free wall due to thrombus coverage and tissue growth may disrupt shear-stress-regulated lumen preservation. This alteration results in increased shear stress, potentially transforming the lesion into a vulnerable plaque prone to rupture.

Plaque rupture provokes the lack of endothelial integrity, which is the main determinant of vascular tone, the activation of inflammation, and the diffusion of molecules to the subendothelial layer. The integrity of the endothelium is maintained by the presence of intercellular junctions (occludin, claudin, junctional adhesion molecules, cadherin, and gap junctions), responsible for the interconnection of endothelial cells, while the integrins link the endothelium to the cellular matrix and subendothelial layers [70]. In terms of integrity, endothelial cells express molecules with antiplatelet, anticoagulant, and fibrinolytic roles.

**Figure 2:**
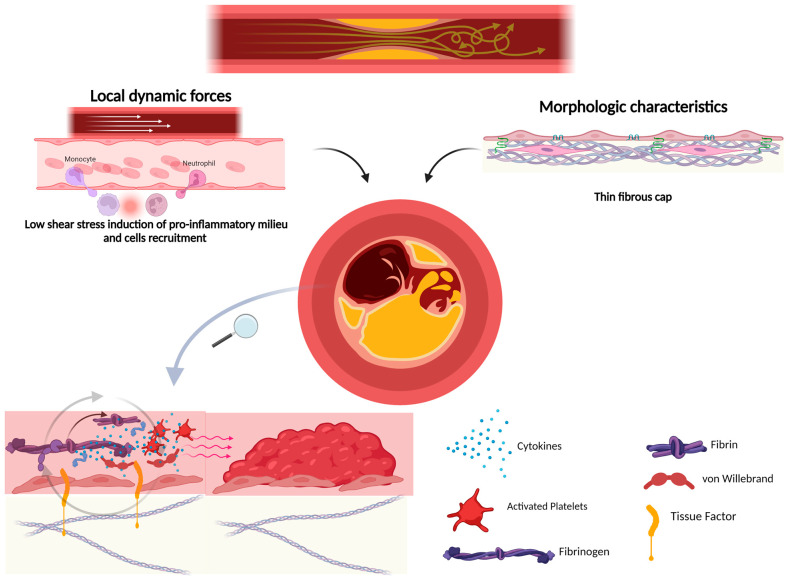
Plaque rupture and thrombosis. Low shear stress triggers the activation of endothelial cells and leukocytes, leading to the upregulation of proinflammatory processes that increase the vulnerability of the lesions. The rupture of the plaque results in compromised endothelial integrity, which is the primary determinant of vascular tone, inflammation activation, and the diffusion of molecules into the subendothelial layer. Upon endothelial damage, platelets adhere to the vascular wall, initiating the formation of a platelet-rich thrombus through the interaction between the platelet glycoprotein (GP) Ibα receptor and von Willebrand factor (vWF), expressed by the injured endothelium. In addition, the GPIbα receptor recruits circulating leukocytes by binding integrins and P-selectin, thereby perpetuating the inflammatory cascade (Created with BioRender.com).

When the endothelium is damaged, platelets adhere to the vascular wall, inducing the formation of a platelet-rich thrombus through the binding of the platelet glycoprotein (GP) Iba receptor and von Willebrand factor (vWF), expressed by the damaged endothelium. The GPIba receptor also recruits circulating leucocytes to bind integrins and P-selectin, perpetuating the inflammatory process [71,72] (Figure 2). Two platelet collagen receptors mediate platelet adhesion and activation [GPIa/IIa (integrin a2b1) and GPVI (immunoglobulin)], favoring the growth of the thrombus [73].

The exposition of subendothelial TF induces the activation of the coagulation system and the production of thrombin, which cleaves fibrinogen into fibrin, which stabilizes the platelets-rich thrombus [71]. As a confirmation of the crucial role of TF, it has been shown that the inhibition of TF with local administration of TF pathway inhibitors blocks atherothrombosis [74]. Moreover, VSMCs release TF-rich microparticles, increasing the expression of this molecule on the damaged surface [75].

Thrombin has a direct role in thrombus growth, inducing, on platelets, the activation of the receptors PAR1 and PAR4 responsible for several intracellular G protein-coupled signaling pathways (Gq, G12/13, Gi, and G2) determining platelet degranulation, activation, and aggregation [76].

Other mediators, such as thromboxane A2 and ADP, are involved in perpetuating platelet recruitment and activation, inducing the progression of the clot. Atherothrombosis is maintained by the activation of all these pathways inducing platelet degranulation and the local accumulation of several molecules, such as ATP, serotonin, Ca^2+^, adhesion proteins (e.g., fibrinogen, fibronectin, vWF, thrombospondin, vitronectin, P-selectin, and integrin aIIbb3) and coagulation factors (e.g., FV, FXI, plasminogen activator inhibitor type 1, plasminogen, and protein S).

Related to this, inflammation is directly responsible for thrombus growth and different cytokines show a direct prothrombotic role, perpetuating the adhesion of platelets on the vascular wall.

One of the molecules involved in atherothrombosis is CRP: when circulating CRP adheres to the platelet surface, it is cleaved into the monomeric form, which exhibits direct prothrombotic activity, favoring platelet adhesion [77].

Also, IL-1-induced inflammation and activation of the inflammasome are responsible for atherothrombosis, as demonstrated by the reduction in cardiovascular events using IL-1 antagonist molecules in the CANTOS study [78].

Recent studies have found a possible role of neutrophil extracellular traps (NETs), fragments of chromatin surrounded by nuclear proteins released from neutrophils, in the link between inflammation and atherothrombosis [79].

Apart from known stimuli such as platelet activation, hyperlipidemia, and oxidized phospholipids, NLRP3 inflammasome activation in neutrophils has recently been found to be related to NETosis.

NET formation is derived from histone citrullination induced by the enzyme peptidylarginine deiminase 4, which is a marker for NET formation [80]. Interestingly, ulcerated and inflamed plaques are associated with a higher neutrophil-to-lymphocyte ratio, a systemic inflammation marker.

Moreover, elevated concentrations of peptidylarginine deiminase 4 have been associated with higher NLR levels, suggesting the possible role of NETs in the instability of atherosclerotic plaques [81].

A recent study showed that the deficiency of ABC transporters in the macrophages of knock-out mice was related to inflammasome activation in macrophages, promoting the accumulation of neutrophils and elevated NETs in plaques due to the accumulation of cholesterol in cells [82,83,84], favoring plaque rupture. In addition, IL-1β intervenes in the cross-talk between macrophages and neutrophils; in fact, the IL-1β-induced NLRP3 inflammasome activation in macrophages increases NETosis, but this process is blocked in cases of NLRP3 inhibition in neutrophils. So, both NLRP3 inhibition and IL-1β inhibition block NETosis and reduce the accumulation of neutrophils on the atherosclerotic plaques, thus representing a potential therapeutic strategy for the future [85].

Plaque rupture represents the final act of a complex molecular mechanism that involves cross-talk between the endothelium, immune-mediated response, and plaque milieu, which can manifest itself as different expressions of vulnerability depending on the patterns involved in this process. Table 1 summarizes the main molecules associated with the individual characteristics of plaque vulnerability that will be discussed in this review. The importance of recognizing the role of these molecules is twofold. First of all, the study of the molecular pathways involved in the development of plaque complications allows us to refine the use of appropriate drugs toward new potential therapeutic targets. Secondly, many of these molecules can represent important biomarkers that can be measured both at the plasma level and through tissue specimens for proteomic analysis, providing complementary information about the activation of the aforementioned pathways.

## 5. Plaque Ulceration

Plaque vulnerability is commonly associated with carotid plaque ulceration, an essential indicator of previous plaque rupture and a strong predictor of future cerebrovascular events. Carotid plaque ulceration or surface irregularity is characterized by a fissure or erosion on the surface of a plaque. Using basic and advanced imaging modalities, the surface of a plaque can be defined as smooth, irregular, or ulcerated. Ulceration is reserved for cavities measuring at least 1–2 mm [86].

Ulceration is a significant factor in vulnerability and is linked to the development of neurologic symptoms on the pathological basis of arterial embolism of thrombotic material. Several studies have demonstrated that ulcerated carotid plaques are more common in patients with anamnestic stroke or transient ischemic attack, and ulceration is more frequently found in patients with symptoms (ipsilateral or contralateral) and is also associated with the occurrence of new symptoms in asymptomatic patients [87].

A complex mechanism is responsible for plaque vulnerability characterized by erosion or ulceration. Inflammation accumulation, proteolytic enzyme release by macrophages, and local hemodynamic factors play a role in the pathogenesis of ulceration [135]. It has been shown that the promoters of the ulceration process are intraplaque inflammation and the activity of a series of MMPs, the primary physiological regulators of the extracellular matrix. The study conducted by Loftus et al. [51] on the expression of MMP-9 within this tissue demonstrated a significant increase in MMP-9 localized in the most unstable carotid plaques and the plaques of more symptomatic patients. Interestingly, MMP-9 has been considered an excellent candidate for future pharmacotherapy intended to stabilize carotid plaques and prevent stroke [89].

The complex mechanism of atherosclerotic plaque formation and rupture primarily involves the interaction of lipids and immune-regulated inflammatory pathways. It has been demonstrated that the CD40–CD40L system could cause plaque instability. Wang et al. showed that CD40L expression on peripheral blood monocytes is associated with carotid atherosclerosis and plaque instability. Furthermore, CD40L expression in circulating monocytes was higher in patients with LAAS, followed by the carotid artery stenosis group [39].

The improvement of proteomics tools has allowed for the unraveling of new circulating markers for atherosclerosis that can be targeted as early diagnostic traits, in preventing such life-threatening events, or as targets for new drug therapies [90]. Martin-Ventura et al. [90] showed that heat shock protein 27 secretion into the culture medium was significantly lower in atherosclerotic plaques compared to control arteries, the plasma level expression was lower in patients with carotid stenosis concerning healthy controls, and the expression of this protein in the unstable human carotid plaque is lower than that in stable plaques. Rocchiccioli et al. verified a significantly higher concentration of thrombospondin-1 and the vitamin D-binding protein in atherosclerotic subjects [136]. Dellas et al. assessed a mouse model for studying plaque ulceration. They showed that the *APO E*^-/-^ mouse model overexpressed the urokinase-type plasminogen activator, suggesting that the loss of basement membrane proteins may play a central role in advanced plaque rupture [137]. Finally, some molecular markers such as vWF and ADAMTS13 are known to induce, respectively, platelet adhesion and aggregation and cleave VWF multimers into smaller sizes. They both have been associated with ischemic stroke, but in a recent study, the VWF antigen level and ADAMTS13 activity seemed not to be related to vulnerable plaque features, such as the volume of IPH and LRNC and plaque ulceration [138].

## 6. Microembolism

Microembolisms from carotid plaques are one of the mechanisms responsible for cerebral ischemia. In 1990, Spencer proved that signals registered in the transcranial Doppler (TCD) monitoring of patients before and during an endarterectomy procedure were assumed to be microemboli from carotid plaques [139].

Microembolic signals (MESs) can also be detected in asymptomatic patients, even if the highest rate of MESs is commonly registered in patients with acute cerebrovascular events and in more than 70% of cases of carotid stenosis [140].

Related to this, MESs may be considered not only markers of plaque instability but also independent risk factors for the incidence and the recurrence of ischemic strokes [141,142]. Even if plaque instability is the primary determinant for microemboli, some specific biomarkers have been associated with a higher risk of distal embolization, supporting the presence of specific mechanisms responsible for this condition.

Commonly, microembolism has been attributed to vulnerable plaque fragmentation [143]; thus, it has been recently proposed that microemboli may derive from platelet aggregation on ulcerated lesions after rupture [144].

Recent studies have supported this hypothesis, showing different protein expression on platelet surfaces in patients with and without MESs. In particular, considering patients with MESs on TCD monitoring, a lower expression of P-selectin on platelets and a higher serum concentration of this protein have been detected in cases of symptomatic and asymptomatic stenosis [145]. On the other hand, a lower expression of thrombospondin has been found in patients with symptomatic stenosis and MESs [145]. Elevated platelet counts and neutrophil–platelet and lymphocyte–platelet complexes were found in early and late symptomatic patients compared with asymptomatic ones, especially if the degree of the stenosis was >70% [146,147,148], suggesting that platelet activation promotes plaque instability through several mechanisms such as enhanced cell adhesion, smooth muscle cell proliferation, and the activation of the coagulation system [149].

These data confirm that platelet activation plays a crucial role in cerebral microembolism, as demonstrated by the efficacy of antiplatelet drugs in reducing the MES rate [150,151,152].

Recently, attention has been turned toward circulating platelet-derived microparticles (PMPs), small vesicles (diameter from 0.05 to 2 mcm) containing phospholipidic compounds from cellular membranes released from platelets. These particles expose antigenic proteins on their surface, which allow for their specific identification in the serum, distinguishing them from other inflammatory cells [153]. PMPs have been related to plaque instability and ischemic cerebrovascular events, suggesting their possible role as a marker for cerebrovascular risk [154]. Furthermore, PMP levels were higher in patients with acute ischemic strokes, while no significant differences were found between symptomatic carotid stenosis of more than 60% and healthy controls, suggesting that platelet microparticles may be correlated with microembolisms. Supporting this hypothesis, a study carried out by Kandiyil et al. [155] demonstrated that PMP concentrations were higher in patients with MESs on TCD and MRI-documented images of acute ischemic events, underlying the relationship between platelet activation and microembolism and the thrombogenic effect of PMPs in the sites of deposition. Interestingly, antiplatelet medications seem to not influence PMP levels.

The neutrophil count is, notably, a marker of atherosclerosis severity and plaque instability [156]. Recent studies have demonstrated that peripheral neutrophilia is associated with a higher risk of microembolism. A higher neutrophil count has been associated with a higher MES rate in patients who have undergone carotid stenting [157]. This association was confirmed in patients with recently symptomatic carotid stenosis without revascularization [158]. On this view, the neutrophil count can be considered a marker of microembolisms, considering the persistent strength of the association even after adjustment for the National Institutes of Health Stroke Scale (NIHSS) and CRP values.

Furthermore, the eC-X-C motif chemokine ligand (CXCL)-16, a chemokine able to recruit and activate T cells (Th1 and Tc1 cells), has recently been associated with acute cerebrovascular disease. CXCL16 is a protein with several functions: it plays an essential role in inflammation, directing leukocyte migration, inducing the secretion of proinflammatory proteins, such as interferon-gamma (IFN-γ) and IL-2, after T cell activation, and acting as a scavenger for oxidized LDL [159]. It is known that CXCL16 is related to atherosclerotic disease severity; in fact, its surface expression on carotid plaques [160] and the serum concentrations of its mRNA [161] are higher in patients with cerebrovascular events compared with asymptomatic plaque. Recent studies have also found a correlation between this protein and microembolization: in fact, higher serum concentrations of CXCL-16 have been detected in patients with MESs on TCD monitoring of LAAS, with a high sensitivity and specificity (88.5% and 56.5%, respectively) for a cutoff value of 2.115 ng/mL [162]. These data suggest the potential role of this chemokine in the pathogenesis of cerebral microembolism.

Moreover, higher plasma fibrinogen concentrations and a decreased ratio of CD4+ CD25-high T reg cells have been found in MES+ compared with MES-patients and carotid atherosclerosis [163], even if more studies need to be carried out to confirm these data.

Finally, regarding the link between inflammation and microembolism, Pini et al. have demonstrated that values of Hs-CRP > 5 mg/L and serum amyloid A > 10 mg/L were associated with a higher MES rate in patients with post-carotid artery stenting asymptomatic cerebral ischemia detected by MRI [164].

## 7. Fibrous Cap Thickness

The term “vulnerable” plaque is frequently used to refer to a lesion with a fibrous cap less than 65 μm thick and infiltrated with macrophages (more than 25 cells per 0.3 mm diameter field). Several studies about plaque rupture suggest that thin-cap atheroma, fibrous caps <65 microns, together with the presence of numerous macrophages within the cap, likely indicate instability and predict plaque rupture [165,166].

As is known, the progression of atherosclerotic lesions occurs through three main stages: an initial, asymptomatic, and non-stenotic stage, followed by an advanced phase where thrombi are formed and cause embolisms. Infiltrated monocytes are recruited to the lesioned area of endothelial cells when modified LDL expresses adhesion molecules and chemokines. When monocytes are differentiated into macrophages, they promote the secretion of connective tissue and the formation of the fibrous cap. If the anti-inflammatory response is not working correctly or is insufficient, atherosclerotic plaques that have been stable for a while may progress and become unstable. The extracellular matrix is hydrolyzed by MMPs and other proteolytic enzymes, causing a loss of thickness of the fibrous cap caused by necrotic macrophages. LDL and modified LDL could be significant targets for preventing the progression and complications of carotid atherosclerosis [167].

The fibrous cap serves as a sub-endothelial barrier, separating the vessel lumen from the atherosclerotic necrotic core [168]. Comprising VSMCs and ECM derived from VSMCs, its primary role is to provide structural support and prevent the exposure of prothrombotic material in the core, thereby averting thrombosis [169]. In response to injury, VSMCs undergo a phenotypic switch to a synthetic state, marked by increased migratory and proliferative activities. Nearby cells initiate the healing process by producing various growth factors, including epidermal growth factor, fibroblast growth factor, insulin-like growth factor, PDGF, TGF-β, and VEGF. In atherosclerosis, stimulated by growth factors from foam cells or endothelial cells (ECs) in the intima, VSMCs from the tunica media migrate to the intima. Macrophage-produced interleukin (IL)-1 enhances PDGF production by VSMCs, leading to autocrine proliferation in the intima. Synthetic VSMCs also increase the production of extracellular matrix components such as interstitial collagen, elastin, and proteoglycans [170]. These proliferating VSMCs, along with ECM production, contribute to the formation of a fibrous cap, crucial for preventing plaque rupture. If mitogen production persists, VSMCs may not revert to the contractile phenotype, facilitating lesion development.

**Figure 3:**
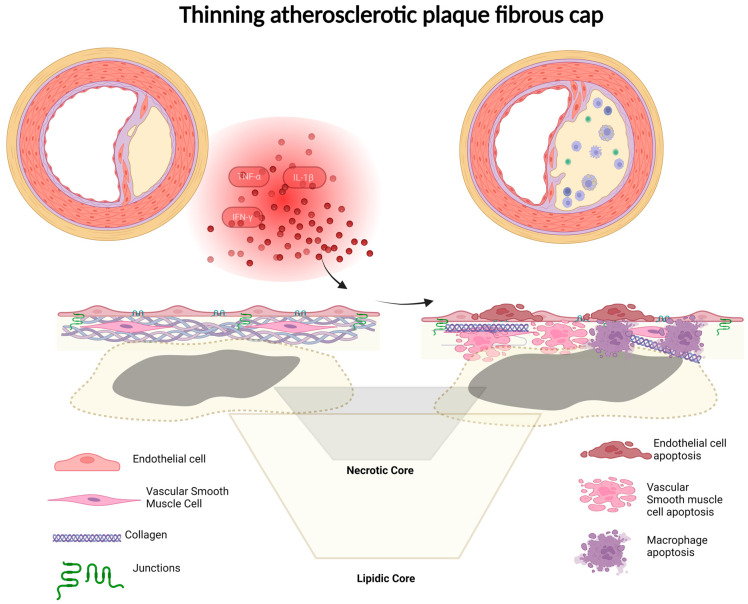
Proinflammatory cytokines such as IFN-γ, TNF-α, and IL-1β promote apoptosis in macrophages and smooth muscle cells, leading to the thinning of the fibrous cap. This thinning allows macrophages to infiltrate the fibrous cap, where they release inflammatory cytokines and MMPs, significantly contributing to the weakening and eventual rupture of the atherosclerotic plaque. The necrosis of the vulnerable plaque occurs due to a combination of macrophage death and impaired phagocytic clearance of apoptotic cells, thereby hastening or triggering plaque disruption through the secretion of inflammatory cytokines and matrix proteases (Created with BioRender.com).

In the fibrous cap, VSMCs and collagen are noted to be present [171], while the necrotic core of the plaque exhibits minimal collagen but a significant amount of free cholesterol. Transcription factors SP1, SP3, and AP1, interacting with the collagen promoter, play a role in the transcription of type I collagen in these cells [172]. The absence of collagen serves as an indication of VSMC loss, rendering the plaque vulnerable [91]. Reduced metalloproteinase activity may contribute to impaired collagen deposition. Collagen is crucial for providing tensile strength to the fibrous cap, and its absence or minimal presence may result in thinning when VSMCs are depleted from the cap, contributing to a rupture with reduced collagen content [173]. The overproduction of VSMCs can lead to plaque growth dependent on angiogenic factors [92]. Type VIII collagen, typically present in small amounts in normal arteries and produced by macrophages and VSMCs, enhances the migration and growth of VSMCs through the extracellular matrix. Fibrous cap characteristics, including thickness, cellularity, matrix composition, and collagen content, play vital roles in plaque stability. The exact mechanism of plaque rupture involves cap thinning, elevated inflammatory cytokines, matrix-digesting proteases, decreased collagen synthesis, and the presence of injured or apoptotic cells within the necrotic core. Cells contributing to plaque formation are implicated in plaque rupture and subsequent thrombosis. Molecular mediators associated with atherogenesis may alter collagen metabolism, thinning or weakening the fibrous cap. Inflammatory cells secrete cytokines, growth factors, TF, IFN-γ, MMPs, and reactive oxygen species. The accumulation of free cholesterol induces the apoptosis of macrophage-derived foam cells, contributing to the formation of the necrotic core. Excess extracellular unesterified cholesterol crystallizes, leading to complicated atheroma and the eventual total occlusion of coronary artery branches. Continued inflammatory responses, driven by proinflammatory cytokines, destabilize atherosclerotic plaques, with IFN-γ, IL-18, growth differentiation factor-15, and TNF-like weak inducer of apoptosis promoting destabilization, while TGF-β causes stabilization [93]. Proinflammatory cytokines such as IFN-γ, TNF-α, and IL-1β stimulate macrophage and smooth muscle cell apoptosis, thinning the fibrous cap (Figure 3). Macrophages infiltrate the thinned fibrous cap, secreting inflammatory cytokines and MMPs, playing a pivotal role in weakening and ultimately rupturing the atherosclerotic plaque. Necrosis of the vulnerable plaque results from a combination of macrophage death and defective phagocytic clearance of apoptotic cells, accelerating or inducing plaque disruption through the release of inflammatory cytokines and matrix proteases. Mechanical stress from the necrotic core on the overlying cap may also contribute to plaque rupture [94].

The generation of PDGF B chains by macrophages, consistently observed throughout all stages of atherogenesis in both human and experimental models [95,174], likely plays a crucial role in recruiting smooth muscle cells to the intima. The macrophage-produced heparin-binding epidermal growth-factor-like growth factor is also implicated in the migration of smooth muscle cells. Nevertheless, the anticipated outcome involves a mosaic of macrophages and smooth muscle cells. The sorting of cells through cell surface cadherins may contribute to this process [175], given that smooth muscle cells, expressing N-cadherin, can engage in homotypic binding, forming strong cell-to-cell interactions that exclude macrophages. Notably, N-cadherin is co-expressed with VE-cadherin on endothelial cells, promoting contact between endothelial cells and smooth muscle cells [96]. The detailed histological characteristics of rupture-prone plaques are extensively addressed in other sections of this volume. In simple terms, plaque rupture tends to occur in cases featuring a large lipid core, a thin cap, and an abundance of macrophages relative to smooth muscle cells [97]. The prevailing perspective suggests that rupture arises from the degradation of an established fibrous cap, a process primarily mediated by macrophages [176]. An alternative interpretation posits that new areas of fatty streaks at the peripheries of existing fibrous plaques simply fail to develop, or have not yet developed, a sufficiently robust plaque cap. Various agents, including prostaglandins that elevate cAMP concentrations [98,177], nitric oxide that raises cGMP levels, interferon-γ, and TGF-β, counteract the effects of smooth muscle cell mitogens [178,179]. For instance, the transfer of the VEGF gene leads to an NO-mediated reduction in neointima formation following cuff-induced injury in carotid arteries. The pathways involved are likely diverse, encompassing the downregulation of early events such as MAP kinase activity and subsequent events, including the expression of cyclin D1 and c-myc [180]. Leukocytes are likely the primary source of these inhibitory agents. Studies by Hansson and colleagues have demonstrated that interferon-γ infusion reduces intimal lesions caused by balloon injury [99], while lesion promotion occurs in lymphocyte-deficient rats, suggesting similar considerations in atherogenesis. If so, the presence of leukocytes in the shoulder regions of advanced plaques may favor the inhibition rather than the proliferation of smooth muscle cells.

In this scenario, the angiotensin-converting enzyme/angiotensin II (ACE/Ang II) system plays a crucial role in forming atherosclerotic lesions. The activation of ACE/Ang II contributes to the formation of a thick fibrous cap. In the early stage of atherosclerotic lesion formation, endothelial cells and macrophages may secrete biologic factors such as ACE/Ang II and induce intimal thickening and the formation of a lipid core. After maturation of the lesion, macrophages may lose the ability to secrete ACE/Ang II, resulting in the thinning of the fibrous cap. A thin fibrous cap and faint expression of ACE/An II were observed in the shoulder lesion of unstable plaques. Plaque rupture may be exacerbated by the absence of correct ACE/Ang II system activity [181].

A study about the correlation of thin fibrous caps possessing adipophilin-positive macrophages and IPH with a high clinical risk for carotid endarterectomy distinguished patients in four groups of risk and demonstrated that plaques in patients in Grade IV (presence of neurological risk) showed more adipophilin-expressing macrophages in the fibrous cap than in those of the other groups. It has been demonstrated that the infiltration of adipophilin-positive macrophages in the fibrous cap may be correlated with the instability of neurological status. In terms of clinical approaches, this evidence invites us to avoid stenting procedures and perform carotid endarterectomy [182].

Recently, research studies have investigated the relationship between small non-coding microRNA expression and carotid plaque development and vulnerability. MiRNAs have been shown to play a crucial role in the atherosclerotic process, which suggests that they could be used as potential biomarkers for the diagnosis and prognosis evaluation of cardiovascular events, as demonstrated by multiple recent studies.

VSMC balance can prevent atherosclerosis. In advanced carotid plaques, the expression of miR-145 and miR-210 drives the migration and proliferation of VSMCs, promoting the stability of the fibrous cap and prevention of plaque rupture. The most studied miRs that can re-establish contractile function in VSMCs and potentially be used as therapeutic targets are miR-22 and miR143/145.

In contrast, the predictor of plaque instability seems to be miR-92a, which regulates MMP-9 overexpression, and miR-200c, which was found to be upregulated in carotid plaques that presented unstable features in imaging studies.

However, studies that have identified specific biomarkers involved in developing a thin fibrous cap are limited, and research is necessary to identify particular microRNAs that could be used in carotid atherosclerosis [183].

Furthermore, finding circulating biomarkers that can identify patients with thin-cap atherosclerosis lesions at a high risk of atherothrombotic stroke remains a challenge [164].

One important issue that is still unresolved is distinguishing between different lesions by the identification of genes specifically associated with rupture. Faber et al. [184] tried to provide a solution, reporting the presence of perilipin exclusively in lesions exhibiting acute rupture. Perilipin was absent in similar vulnerable lesions with necrotic core areas that appeared indistinguishable from those in ruptured lesions expressing the molecule. Consequently, these researchers may have uncovered a potentially significant causative factor for rupture, or alternatively, one among several molecules expressed post rupture. It is improbable that perilipin serves as the exclusive indicator of rupture. Presenting a single gene among the 35,000 candidates possibly implicated in this process is, at most, an initial stage. While the era of plaque imaging is emerging, the ability to discover individual or even a few new genes, as exemplified in the current study, is approaching its realization. Current estimates suggest approximately 35,000 sites of RNA expression in the genome. The technology for representing all these sequences in a hybridization array is already nearly available in the existing generation of microarrays. In theory, one could extract RNA from both a ruptured plaque and a non-ruptured plaque, utilizing a large-scale expression array to meticulously outline all potential differences in expression. Single observations, such as those in the present study, will eventually be considered anecdotal as this technology progresses.

In another recent study [100], Bazan et al. compared the transcriptomes of asymptomatic and recently ruptured carotid plaques to determine the molecular mechanisms active at the time of rupture. The authors found that atherosclerotic plaques that have recently ruptured show heightened activity in proinflammatory genes responsible for attracting and mobilizing leukocytes to the vessel wall. In the recently ruptured group, there was an increase in the expression of XCR1, a chemokine receptor, and CD177, which facilitates neutrophil activation. Furthermore, the same group exhibited elevated levels of various transcripts linked to B cell function, including numerous immunoglobins, as well as transcripts associated with B cell proliferation (MZB1) and activation (CD79A, SH2D3C, and ZAP70).

The samples of recently ruptured atherosclerotic plaques also displayed alterations in transcript expression, potentially linked to the thinning of the fibrous cap, characterized by reduced VSMC proliferation and migration and increased apoptosis. SDC1 and SIK1, associated with decreased VSMC proliferation and migration, were among the genes showing changes. In addition, there was an upregulation of transcripts related to the inhibition of cell proliferation (NPDC1, DOK3, and ZBTB17), and a decrease in the expression of a pro-migration transcript (TMSB15B) was observed in the recently ruptured samples. Numerous transcripts encoding Ras GTPase-activating proteins (RASA4, RASA4B, ARHGAP4, HMHA1, and SH3BP1) were identified, promoting the inactivation of Rac1 and cdc42, thereby inhibiting proliferation and migration. Finally, in a fascinating study by Bobryshev et al. [185], a comparison between the relative number of calcified matrix vesicles in fibrous caps of vulnerable (FCT < 100 μm) and presumably stable atherosclerotic plaques (FCT > 100 μm) was performed. The quantification of matrix vesicles in stable and vulnerable plaques showed that the relative densities of matrix vesicles were significantly higher in fibrous caps of vulnerable plaques than those in stable plaques. These results suggest that the accumulation and calcification of matrix vesicles could be involved in the process of plaque rupture, possibly through the alteration of the texture of connective stroma in plaque fibrous caps.

## 8. Neoangiogenesis and Intraplaque Hemorrhage

The regulation of angiogenesis in atherosclerosis involves molecular mechanisms primarily driven by oxygen deficiency [186]. Hypoxia induces monocyte/macrophage survival and the uptake of ox-LDL by macrophages [187], while also upregulating MMP expression in various plaque cells, contributing to plaque instability [188].

Angiogenic sprouting entails the proliferation and migration of ECs into avascular areas. Pathological angiogenesis is a consistent feature of atherosclerotic plaque development, and its progression is linked to a gradient of VEGF triggering EC growth from existing adventitial vasa vasorum [189]. However, the precise origin of plaque neovessels remains incompletely established [190].

Neoangiogenesis is intimately linked with plaque advancement and likely serves as the primary origin of IPH at locations with microvessel incompetence. Focal aggregations of angiogenic factors derived from T cells and macrophages contribute to (1) the branching of vasa vasorum around the necrotic core; (2) the formation of immature vessels; and (3) the loss of the basement membrane around functional capillaries. This sequence of events initiates the leakage of red blood cells (RBCs) into the plaque and sets off a cycle of inflammation and neovascularization.

**Figure 4:**
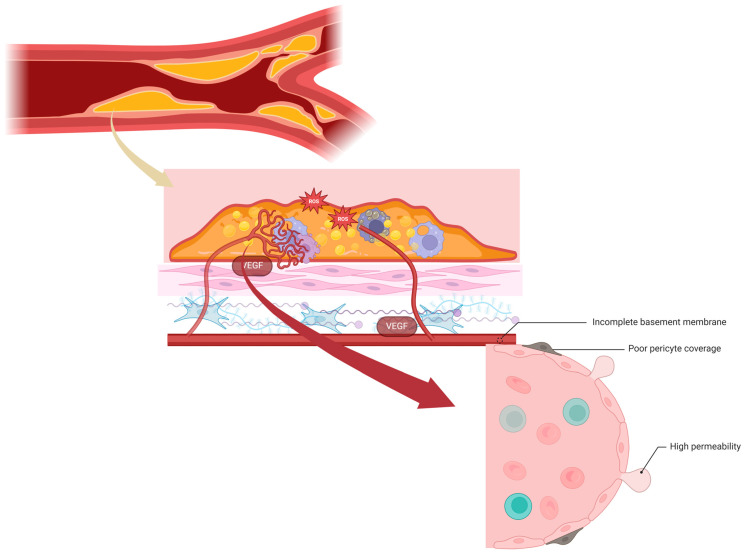
Neovascularization in vulnerable plaque. Pathological angiogenesis consistently accompanies the development of atherosclerotic plaques, with its progression correlated to a gradient of VEGF that triggers endothelial cell growth from pre-existing adventitial vasa vasorum. Neovascularization in vulnerable plaques demonstrates immaturity, irregularity, and fragility due to compromised structural integrity. These vessels exhibit a discontinuous basement membrane and a low number of tight junctions between endothelial cells. These immature vessels have limited pericyte coverage and are susceptible to the leakage of circulating cells, which can lead to intraplaque hemorrhage (Created with BioRender.com).

Neovessels in vulnerable plaques exhibit immaturity, irregularity, and fragility due to compromised structural integrity [101]. They possess a discontinuous basement membrane and a low number of tight junctions between ECs [191]. Additionally, these premature vessels have limited pericyte coverage and are prone to the leakage of circulating cells, leading to IPH [192]. Similar characteristics are observed in newly formed vessels in tumors, which are heterogeneous, organized chaotically, lack hierarchical branching, and display hyper-permeability [102] (Figure 4).

In advanced atherosclerotic lesions, neovessel leakage becomes the primary entry point for inflammatory cells. RBCs facilitate the extravasation of circulating inflammatory cells by increasing the number of rolling and adhering monocytes, potentially enhancing the force and frequency of collisions with the endothelium [193]. Neutrophils and mast cells, associated with neovessels, release granular content rich in serine proteases and MMPs, contributing to elastic fiber and basement membrane degradation [194]. This high proteolytic activity can lead to fibrous cap thinning and plaque erosion [195].

Moreover, RBC influx and lysis increase the demand for macrophage activity to phagocytose RBC remnants. Combined with impaired efferocytosis in atherosclerotic lesions, hindering the clearance of apoptotic cells by lesional macrophages, this accumulation may explain why macrophages accumulate in the atherosclerotic necrotic core, potentially exacerbating vascular inflammation [196].

Basic fibroblast growth factor (bFGF) is also involved in promoting intraplaque angiogenesis, and it is stimulated by local hypoxia, leading to the formation of neovessels from the vasa vasorum in the adventitia [197]. These newly formed vessels are immature, lacking proper pericyte coverage and tight junctions between endothelial cells, resulting in IPH. This hemorrhage, characterized by the extravasation of RBCs and inflammatory cells, sustains ongoing inflammation. Simultaneously, invading inflammatory cells, especially macrophages, stimulate the synthesis of various angiogenic factors, further promoting plaque angiogenesis. bFGF also facilitates macrophage infiltration into lesions by inducing the expression of chemokines and adhesion molecules [198]. The accumulation of foam cells, derived from macrophages, contributes to lipid storage, atherosclerotic plaque growth, and a proinflammatory plaque phenotype. This interplay between intraplaque angiogenesis and inflammation forms a vicious cycle, driving the atherosclerotic plaque toward an unstable phenotype and potential rupture [103].

bFGF has long been recognized for its role in promoting SMC proliferation and migration, making it a key target in preventing post-interventional intimal hyperplasia [199]. SMCs play a dual role in atherosclerotic lesion stability, not only contributing to lesion formation through intimal proliferation but also playing a crucial role in forming the fibrous cap that covers the lesion. Following vascular injury or endothelial cell activation, SMCs transition from a contractile to a proliferative phenotype, marked by increased cell proliferation and migration. SMCs in the fibrous cap primarily exhibit a contractile phenotype, and the thickness of the cap is critical for plaque stability, as thinning increases the risk of plaque rupture [200]. A higher expression of bFGF in SMCs is observed in unstable plaques compared to stable plaques, and it is known to stimulate a proliferative phenotype [199]. A recent study hypothesized that blocking bFGF signaling could potentially reduce intraplaque angiogenesis, macrophage infiltration, and SMC proliferation, resulting in the stabilization of atherosclerotic plaques, and demonstrated that K5-mediated bFGF signaling blockade in hypercholesterolemic apolipoprotein E (ApoE)3*Leiden mice reduces intraplaque angiogenesis, hemorrhage, and inflammation [201].

A primary contributor to micro- and macrohemorrhages within atherosclerotic lesions is an underdeveloped network of blood vessels that emerges within the intima of a plaque. While the majority of these newly formed vessels have endothelial coverage, they seldom feature mural pericytes or smooth muscle cells.

In the context of atherosclerosis, the permeability of the intraplaque vasa vasorum, promoting leakage, has been characterized through ultrastructural visualization, revealing defects between endothelial junctions [104]. Alternatively, the identification of perivascular vWF immunoreactivity around vessels has been utilized as an indicator of a less intact or leaky endothelium [202]. The leakiness of these vessels is influenced by factors that induce vessel growth, with VEGF and its related receptors and cofactors serving as the principal regulators of vascular permeability in pathologic angiogenesis. The VEGF signaling pathway involves an interaction between growth factor receptors and specific integrins [105]. The VEGF receptors are likely to play a crucial role in cell-to-cell adhesion and communication by directly interacting with cell-to-cell adhesion molecules.

Observational investigations into the progression of the necrotic core highlight IPH as a crucial factor in the growth and destabilization of atherosclerotic plaques. The swift accumulation of erythrocyte membranes brings about a sudden alteration in plaque composition, characterized by the augmented presence of free cholesterol within the core and the excessive infiltration of macrophages.

Examinations of unstable lesions through pathological analysis have revealed an association between IPH, plaque rupture, and an elevated microvessel density. The precise mechanism by which RBCs leak into the necrotic core remains poorly understood. Our research laboratory has documented widespread perivascular staining of vWF within the vasa vasorum of plaques, along with evidence of erythrocyte membranes within necrotic cores. This observation suggests that microvascular disruption or permeability may contribute to lesion progression by providing cholesterol derived from erythrocytes. Apart from a leaky vasa vasorum, the accumulation of erythrocytes can also result from plaque fissuring; a phenomenon described in the coronary vasculature of individuals who succumbed to sudden coronary death [106].

The count of the vasa vasorum was found to be twofold higher in vulnerable plaques and up to fourfold higher in ruptures compared to stable plaques with significant luminal narrowing [203]. Increased T cell numbers are commonly observed at breaks in the medial wall and the base of the necrotic core, as opposed to other plaque regions. It is plausible that T helper cell-driven immune responses, possibly mediated by interferon-γ, may hinder smooth muscle cell proliferation, contributing to medial disruption and the absence of smooth muscle cells in penetrating neovessels.

Recent attention has been directed towards VE-cadherin in carotid atherosclerotic plaques due to its recognized significance in angiogenesis. The VE-cadherin/catenin complex is implicated in the organization and maturation of neovessels and in safeguarding endothelial integrity [204]. Recent evidence, consistent with previous findings by Bobryshev et al. [205], suggests that VE-cadherin is selectively deposited in neovessels surrounded by inflammatory cells, underscoring its central role in this process. Complicated plaques exhibit heightened expression of VE-cadherin, along with the analogous expression of beta-catenin, moderate expression of alpha-catenin, and little to no expression of plakoglobin. The reported necessity of plakoglobin for the formation of ‘mature’ junctions [204] suggests that the absence of plakoglobin in neovessels compromises junction function, increasing vascular permeability.

Significantly elevated expression of VE-cadherin has been demonstrated in complicated plaques, with even higher expression in high-grade stenotic lesions. This finding suggests that VE-cadherin may also play a crucial role in the progression of carotid lesions. Moreover, the strong expression of this molecule in patients with cerebral infarction and clinical symptoms indicates an association between neovascularization and symptomatic carotid disease, as corroborated by previous reports [206]. Consequently, VE-cadherin could be considered a marker of advanced carotid atherosclerotic plaques [107].

Recently, some researchers employed an innovative translational approach in proteomics [108] to unveil signature molecules associated with the development of carotid atherosclerosis. A captivating study also integrating transcriptomics discovered an abundance of biliverdin reductase B in both plaque tissue and plasma. This association was linked to IPH and processes that were previously correlated with plaque instability [207].

Although the mechanisms underlying plaque activation have not been completely elucidated, recent data suggest that macrophages and smooth muscle cells release angiogenic factors [208], causing the migration and proliferation of endothelial cells in the intima and the expression of VE-cadherin [209]. VE-cadherin is involved in the maturation, expansion, branching, and remodeling of endothelial cells into a network of new blood vessels in plaques [210]. Neovascularization serves as the primary route for inflammatory cells migrating into the intima, involving cell adhesion molecules (E-selectin, intercellular adhesion molecule-1, and vascular cell adhesion molecule-1) [211].

Moreover, a recent study underlined the potential role of Sema7A in promoting VEGFA/VEGFR2-mediated neovascularization in *ApoE^-/-^* mice, supporting a pivotal role of Sema7A in the progression of human atherosclerosis and IPH [212].

Finally, according to recent data, the advancement of IPH is linked to the involvement of endogenous IL-1β, which inhibits physiological angiogenesis in the atherosclerotic plaque, ultimately resulting in the development of permeable neovessels. In addition, the induction of MMP-9 expression may play a role in the formation of these leaky neovessels [109].

## 9. Lipidic Core

Research on the lipidic core of atherosclerotic plaques is a subject of considerable importance in understanding plaque vulnerability, prognosis, and developing targeted therapies, as its characteristics play a crucial role in determining plaque stability. The presence of a large lipid core occupying more than 40% of the plaque area is considered one of the main criteria for plaque vulnerability [10,212]. Furthermore, previous studies have separated the resulting plaques by stability and measured the lipid subcomponents using mass spectrometry, showing that the lipid content of unstable plaques was higher than that of stable plaques.

The pathogenesis of this more clinically significant plaque component remains controversial. Lipids, primarily LDL carried by blood, may undergo direct accumulation within the extracellular space or be internalized by macrophages, likely through scavenger receptors following oxidative modification [110]. Subsequently, accumulation may occur indirectly through the necrosis of lipid-filled macrophages, also known as foam cells [111]. Nevertheless, findings have challenged the notion that the core primarily originates from deceased foam cells in the superficial intima (fatty streaks). Instead, evidence suggests that the core develops predominantly from a gradual buildup of lipids in the extracellular matrix of the deep intima, resulting from intricate interactions between insulating LDL and glycosaminoglycans, collagen, and/or fibrinogen.

The lipid core within a plaque is devoid of supporting collagen, is avascular, hypocellular (except at the periphery of the core), and rich in extracellular lipids. By quantitatively analyzing lipid plaque composition, it has been determined that, in plaques, the cholesteryl ester content is the highest, followed by phospholipids, then by free cholesterol and triglycerides [213].

A genome-wide microarray expression study in advanced carotid plaques showed that in the transition from being asymptomatic to causing symptoms, there is a concurrent increase in the expression of macrophage adipophilin, a marker indicating the loading of lipids [214]. Moreover, the overexpression of the CD36 and ATP-binding cassette (ABCA) 1 genes in CPs was identified. CD36 and ABCA1 appear to have a crucial role in regulating the intake and release of lipids by macrophages. CD36, known for its role in scavenging oxidized LDL, is a multifunctional membrane glycoprotein considered vital for the formation of foam cells. Its expression is further enhanced by its ligand, creating a potentially vicious cycle leading to excessive intake. However, the detrimental effects of this cycle are mitigated to some extent by ABCA1, a membrane transporter protein belonging to the ABC family. ABCA1 is widely recognized for its atheroprotective properties, as it helps in removing free cholesterol and phospholipids. Additionally, it plays a crucial role in initiating the reverse transport of cholesterol in macrophages, a process essential for maintaining cholesterol homeostasis [112]. Mutations in ABCA1 can lead to a severe deficiency in high-density lipoprotein (HDL), causing cholesterol buildup in tissue macrophages and promoting atherosclerosis. Also, ABCG1 plays a crucial role in the efflux of cellular cholesterol to HDL and its apolipoproteins. Furthermore, both ABCA1 and ABCG1 influence the release of inflammatory signals from cells by adjusting the amount of cholesterol in the cell’s outer layer and inside its compartments, reducing inflammatory stress [113]. Metabolites elevated in metabolic syndrome and diabetes negatively impact ABCA1, reducing cholesterol export from macrophages. In addition, oxidative modifications of HDL in cardiovascular disease patients hinder apolipoproteins from efficiently removing cellular cholesterol via the ABCA1 pathway. These findings suggest that a compromised ABCA1 pathway may contribute to increased atherogenesis in common inflammatory and metabolic disorders, making it a crucial target for potential cardiovascular disease therapies [215].

Other specific biomarkers associated with the lipidic core content, such as elevated levels of lipoprotein-associated phospholipase A2 (Lp-PLA2) and lysophosphatic acid, have been studied for their prognostic role in the prediction of cerebrovascular accidents. PLA2 is associated with Lp(a). Lp(a) is a type of lipoprotein that promotes the development of arterial plaques and is found only in areas with atherosclerosis. Pathological evidence indicates that Lp-PLA2, associated with Lp(a), might contribute to atherosclerosis; in more advanced plaques, there is strong Lp-PLA2 staining in areas rich in lipids and oxidation byproducts [216,217]. In relation to the lipidic core content, the determination of Lp-PLA2 could be used in conjunction with clinical evaluation to assist in predicting a patient’s risk of cardiovascular disease [218].

Lysophosphatidic acid (LPA) builds up in the lipid-rich core of carotid atherosclerotic plaques and is released when the plaque ruptures. This release activates platelets and can lead to the formation of blood clots within the artery. In human atherosclerotic plaques, LPA levels are significantly higher (13-fold) compared to those in normal arterial tissue, and the highest concentrations are found in the lipid-rich core. The elevated LPA concentration in the plaque’s lipid core may be exposed after cap rupture, potentially causing the formation of local blood clots. The LPA concentrations were significantly higher and more common in patients with unstable plaques. This means that LPA determination may be a useful biomarker in the clinical identification and prediction of unstable plaques and the guiding of treatment [219].

The Multi-Ethnic Study of Atherosclerosis showed that in people with thickened carotid walls, the plasma total cholesterol level was strongly associated with lipid core presence detected by MRI and, as a consequence, high total cholesterol levels may be associated with the rupture proneness of atherosclerotic lesions in the general population [220], confirming the pivotal role of targeted therapy with statins and lipid-lowering drugs.

Innovative experimental models are shedding light on the intricate process of cholesterol-rich vesicle formation, particularly evident in animal models such as rabbits, where cholesterol feeding initiates the deposition of vesicular lipid deposits beneath the arterial endothelium [221]. In addition, researchers like Mitchinson et al. suggest that vesicles observed in human atherosclerosis might comprise ceroid, a complex of lipid and protein formed through peroxidation and cross-linking, potentially generated by macrophages exposed to LDL or polyunsaturated cholesteryl esters [222]. However, quantification is necessary as early estimates indicate ceroid may represent only a small fraction of core lipids compared to cholesterol-rich vesicles [221].

Further insights into cellular mechanisms come from Tangirala et al., who investigate how lysosomal hydrolysis of cholesteryl esters contributes to free cholesterol accumulation within cells. Their experiments demonstrate that macrophages rapidly accumulate free cholesterol in lysosomes via hydrolysis, leading to the formation of cholesterol crystals within lysosomes, with implications for cholesterol-rich core initiation in human atherosclerosis [223]. In addition, proposed mechanisms for extracellular vesicle formation include selective or nonselective cell death and the extrusion of lysosomal contents, as suggested by Schmitz, Robenek, and colleagues [98]. Chung et al. propose a novel mechanism suggesting extracellular vesicles are formed through the interactions of lipoproteins, contrary to the common assumption of LDL involvement. Supporting their hypothesis, cholesterol-rich vesicles from the human aorta contain various apolipoproteins but minimal apoB, indicating a potential alternative pathway for vesicle formation [114]. Oxidized lipid derivatives play a significant role in atherosclerosis progression. Brooks et al. identified oxidized derivatives of cholesterol and cholesteryl esters within human atherosclerotic lesions, subsequently discovering oxidized LDL within fibrous plaques. Myeloperoxidase, an oxidative enzyme, is detected in both the shoulder regions and the core of plaques, highlighting a connection between LDL oxidation and plaque aggregation [221]. Carpenter et al. investigated oxidized lipids in advanced human aortic plaques, revealing the presence of hydroperoxy and hydroxyoctadecadienoic acids derived from lipid peroxidation, alongside low concentrations of oxysterols compared to in vitro models. Interestingly, enzymatically oxidized cholesterol derivatives were more abundant in core lipids, suggesting potential differences in bioactivity [115,224]. Cellular responses to lipid accumulation and complement activation within atherosclerotic lesions are also crucial. Complement components are abundant in the core, potentially generating toxic responses. Antigenic markers of complement activation are identified in the atherosclerotic core, indicating potential implications for lesion development [225,226]. The potential for excessive cholesterol buildup within cell membranes near the core necessitates evaluation. While cellular defense mechanisms exist, including increased sphingomyelin synthesis, their failure may lead to membrane function derangement and cellular toxicity [227,228]. In addition, the impairment of reverse cholesterol transport in atherosclerotic tissue remains a plausible but unproven hypothesis requiring further examination. A comprehensive understanding of cellular mechanisms and lipid interactions within atherosclerotic lesions is crucial for developing targeted therapies. Future research should focus on elucidating the intricate pathways involved and their implications for disease progression and treatment.

The persistent inflammatory response ultimately destabilizes atherosclerotic plaques through the action of proinflammatory cytokines. Studies have demonstrated that IFN-γ, IL-18, growth differentiation factor-15, and TNF-like weak inducers of apoptosis contribute to plaque destabilization while TGF-β promotes stabilization [116]. IFN-γ, TNF-α, and IL-1β promote the apoptosis of macrophages and foam cells, leading to lipid core enlargement. Moreover, these cytokines induce the apoptosis of SMCs, resulting in the thinning of the fibrous cap [173]. Proinflammatory cytokines also impede the synthesis of plaque-stabilizing ECM components produced by SMCs. For instance, IFN-γ inhibits collagen synthesis by SMCs [116].

Macrophages infiltrate the thinned fibrous cap, expressing and secreting numerous inflammatory cytokines and MMPs that digest the stabilizing matrix, playing a pivotal role in weakening and ultimately rupturing the atherosclerotic plaque. It has been reported that the necrosis of the vulnerable plaque is attributed to a combination of macrophage death and defective phagocytic clearance of apoptotic cells, accelerating or inducing plaque disruption by releasing inflammatory cytokines and matrix proteases [168]. Finally, the mechanical stress caused by the necrotic core on the overlying cap is also considered an important element that can contribute to plaque rupture [94].

## 10. Microcalcification

The process of atherosclerosis, characterized by the accumulation of plaques in arterial walls, is a complex and dynamic phenomenon influenced by various factors, including inflammation and calcification [117,229]. Understanding the interplay between these processes is crucial for unraveling the mechanisms underlying plaque vulnerability and stability.

Recently, there has been growing interest in understanding the impact of calcification on plaque vulnerability [230,231]. According to the classification proposed by Naghavi et al., plaque microcalcifications are considered a minor criterion for determining plaque vulnerability, corresponding to a type Vb lesion as per the American Heart Association’s histological classification [10,232].

The role of calcification in determining plaque vulnerability remains a topic of ongoing debate. Notably, mechanical experiments conducted on human carotid plaques by Mulvihill et al. [233] suggested that calcification within the tissue structure might contribute to the increased vulnerability of the plaque. Conversely, findings by Shaalan et al. [234] indicated that symptomatic plaques tend to be less calcified and more inflamed than asymptomatic plaques, suggesting a potential reduction in the risk of plaque rupture associated with calcification. Interestingly, a fascinating study found a complex relationship between calcification, intraplaque hemorrhage, and the lipid core within the carotid atherosclerotic plaque. According to their results, plaques with a higher amount of calcification more often contain hemorrhagic components, but less often lipid cores, suggesting that in both small and large plaques, intraplaque calcification may not be a stabilizing factor per se [235].

In a study by Wahlgren et al. [236], thirty carotid endarterectomy plaques were classified as noncalcified and calcified, yielding results consistent with the notion that fibrous cap inflammation is more likely to occur in noncalcified plaques compared to calcified ones. This observation implies a protective effect of plaque calcification against rupture.

Computational investigations on microcalcifications have also contributed to our understanding of plaque vulnerability. Kelly-Arnold et al. [237] examined the spatial distribution, clustering, and shape of different microcalcification sizes in fibrous caps, revealing that nearly all fibrous caps contain microcalcifications, but only a small subset has the potential for rupture. Engrenyuk et al. [238] explored stress distribution using multilevel micro-CT-based 3D numerical modeling techniques. Their results indicated that the presence of calcifications (inclusions) increases the peak circumferential stress, with elongated microcalcifications potentially further elevating stress levels. In contrast, macrocalcifications in cap shoulders were found to enhance plaque stability.

Microcalcifications, initially smaller than 50 μm, typically emerge from the lipid pool and early necrotic core, with potential formation in the fibrous cap [117,229]. These microcalcifications can progress into larger structures, such as calcified sheets and nodules, ultimately leading to plaque ossification, which is prominently observed in peripheral arteries [117]. Recent evidence suggests that heavily calcified plaques, characterized by sheets of calcification or ossification, exhibit stability, contrasting with plaques featuring small and diffuse calcifications [239,240]. Intravascular imaging techniques, such as intravascular ultrasound and optical coherence tomography, have revealed that vulnerable plaques are often associated with spotty calcifications, whereas larger calcium deposits may indicate stability [241].

The paradigm of heavily calcified plaques being stable is further supported by the impact of statins on plaque development. Statins, known for reducing adverse cardiovascular events, have been shown to decrease the plaque surface while increasing calcification, as demonstrated by IVUS imaging studies [242,243]. This observation has been corroborated by computed tomography and magnetic resonance imaging [244]. Experimental studies in ApoE-deficient mice treated with pravastatin showed the coalescence of microcalcifications into larger structures [245].

The mechanical impact of macrocalcifications on plaque stability appears to be stabilizing rather than harmful, in contrast to the potential danger posed by microcalcifications smaller than 50 μm [246]. Microcalcifications in the fibrous cap, particularly those exceeding 10 μm, have been suggested to exert dangerous mechanical stress, contributing to plaque rupture [233]. Moreover, microcalcifications may not only pose a risk through mechanical stress but also by triggering or amplifying plaque inflammation. Studies using 18F-sodium fluoride PET/computed tomography have associated microcalcifications with macrophage infiltration and apoptosis in carotid plaques, emphasizing their potential role in inflammatory processes [247]. The presence of coronary microcalcifications has been linked to macrophages, further supporting the connection between inflammation and microcalcification [248]. Interestingly, as plaque calcification progresses, macrophage infiltration decreases, and plaques become less symptomatic [249].

The initiation and evolution of microcalcifications towards larger, potentially more stable structures involve cellular and molecular mechanisms. Inflammation has been implicated as a trigger for microcalcification, supported by clinical studies and animal models [250]. Cell necrosis, especially within the necrotic core, serves as a potent inducer of pathological calcification [251]. Compromised macrophage phagocytosis of apoptotic bodies, a hallmark of atherosclerosis plaques, may contribute to the formation of early microcalcifications [192]. Additionally, a form of programmed cell necrosis called necroptosis has emerged as a key player in advanced human plaque development, particularly in macrophages [252].

Inflammatory cytokines, including TNF-α, IL-1β, and IL-6, stimulate VSMCs to transdifferentiate into chondrocyte-like cells, promoting calcification [253,254,255]. Understanding these processes is critical, as inflammation appears to precede calcification, and the interaction between inflammation and calcification creates a feedback loop that amplifies both phenomena [118,119].

Repair mechanisms associated with calcification play a role in the terminal phase of atherosclerosis, with large and stable calcification deposits contributing to the resolution of inflammation [120]. Macrophages and VSMCs generate calcification inhibitors, such as matrix Gla protein (MGP), Gla-rich protein, and OPN, to prevent additional crystal growth. Furthermore, VSMCs produce bone morphogenetic protein 2 (BMP2), potentially activating the transition of VSMCs into chondrocytes and creating a cartilage-like environment, ensuring the stability of the calcified tissue [256].

TNF-α, among other proinflammatory cytokines, has the potential to stimulate VSMCs to mineralize independently of their transdifferentiation into mineralizing cells. For example, TNF-α reduces the expression of the inorganic pyrophosphate transporter ANKH and diminishes inorganic pyrophosphate export in VSMCs, a condition linked to calcification, as ANK deficiency in mice leads to soft tissue calcification [257]. Furthermore, TNF-α decreases extracellular inorganic pyrophosphate levels through tissue-nonspecific alkaline phosphatase activation, and it is noteworthy that tissue-nonspecific alkaline phosphatase has roles beyond inorganic pyrophosphate removal in mineralized tissues, including functions in inflammation [258].

Tissue-nonspecific alkaline phosphatase expression is induced by TNF-α or IL-1β in human mesenchymal stem cells, leading to increased mineralization, while simultaneously inhibiting the expression of RUNX2 and its transcriptional targets [121]. In addition, TNF-α may induce calcification in VSMCs by downregulating another crucial calcification inhibitor, MGP [122]. Calcium phosphate crystals smaller than 1 μm activate macrophages, triggering the release of proinflammatory cytokines, such as TNF-α, IL-1β, and IL-8 [123]. Macrophages phagocytose these crystals, with the smallest ones being most potent at stimulating TNF-α secretion, suggesting an increase in TNF-α production after internalization. Although the precise mechanisms through which crystals activate TNF-α remain unclear, the activation of IL-1β secretion is better understood in terms of through the NLRP3 inflammasome [119].

Macrophages stimulated by crystals may activate VSMCs through the release of inflammatory mediators, and the potential stimulation of VSMCs to transdifferentiate through the release of receptor activator of NF-κB ligand (RANKL) is a question warranting investigation. The inhibition of RANKL–RANK signaling with OPG has been associated with reduced plaque calcification [259]. VSMCs, although not specialized for secreting proinflammatory molecules, may release cytokines in response to microcalcifications. VSMCs release IL-1β in the presence of calcium phosphate crystals, and this release increases as VSMCs progress toward senescence, a phenomenon observed frequently in human plaques. IL-1β release in VSMCs relies on crystal endocytosis and caspase-1 activity, but not necessarily on NLRP3 activation, as other treatments activating NLRP3-dependent IL-1β secretion in monocytes/macrophages fail to induce IL-1β release in VSMCs [260]. The long-term treatment of human VSMCs with calcium phosphate crystals induces crystal endocytosis and cell death [124]. VSMC apoptotic death often leads to secondary necrosis and the release of the alarmin IL-1α, exacerbating plaque inflammation, while apoptotic bodies from dying VSMCs may serve as a nidus for new calcification, further amplifying inflammation [261].

Recent findings indicate that monocytes and macrophages can produce γ-carboxylated MGP and Gla-rich protein. Both MGP and Gla-rich proteins act as inhibitors of vascular calcification and their production is augmented in the presence of inflammatory molecules or hydroxyapatite crystals [120]. This may contribute to the correlation between the presence of microcalcifications, macrophages, and carboxylated MGP observed in human atherosclerotic plaques [262]. In addition, VSMCs produce OPN in response to calcium phosphate crystals [263]. Phosphorylated OPN, an inducible inhibitor of ectopic calcification, binds to crystals and inhibits their growth. OPN stimulates macrophages to phagocytose crystals, similar to its role in stimulating osteoclasts for bone resorption. Notably, OPN deficiency in atherosclerotic mice leads to the development of more calcified plaques [264].

Many studies have investigated the development of calcification formation and have identified the molecules OPN and osteoprotegerin (OPG) as playing a crucial role in promoting atherosclerosis and modulating vascular mineralization [265,266,267].

OPG, a TNFR family member, can inhibit bone resorption and suppress immune responses. In terms of cardiovascular health, OPG plays a crucial role in preventing the development of atherosclerosis. It is synthesized by macrophages and expressed by both endothelial and smooth muscle cells. Additionally, OPG is present in Weibel–Palade bodies and is released into the extracellular environment when the endothelium is activated. OPG safeguards the endothelium from the apoptotic process and binds to TSP-1 to regulate vascular damage and thrombus formation. Furthermore, in the medial layer, OPG prevents the calcification of the ECM without dissolving the calcium that has already been deposited [125,268,269,270].

As already underlined, OPN is a proinflammatory cytokine with the ability to bind hydroxyapatite crystals to osteoblasts and is involved in systemic inflammatory and remodeling processes. Fibroblasts and macrophages synthesize this protein. Several studies have highlighted a strong correlation between plasma levels of OPN and atherosclerotic lesions, particularly affecting large vessels [126,127].

OPG and OPN co-exist in carotid atherosclerotic plaque demonstrating a modulatory role in inflammatory and calcification processes. OPG is strongly expressed in stable, calcified plaques, while OPN is poorly expressed in calcified plaques and plaques without signs of vulnerability [36].

According to research, both OPN and OPG contribute to inflammation in atherosclerotic cardiovascular diseases and interact with vascular calcification. However, more research is required to fully understand the mechanisms involved. The findings of these studies need to be validated further.

The progression from a disorganized, proinflammatory tissue containing apatite crystals to the formation of new mineralized tissue may not only be exclusive to atherosclerosis plaques but also occurs in bone repair following a fracture. Fracture healing commences with an initial inflammatory phase, crucial for inducing subsequent ossification [128]. The absence of TNF-α, in particular, delays ossification, partly by impeding chondrocyte differentiation [129]. Intriguingly, TNF-α may primarily act through BMP2, as it stimulates BMP2 expression in chondrocytes [271] and endothelial cells [272], and BMP2 deficiency halts the early steps of fracture healing [273]. Consequently, the inflammatory impact of apatite crystals in atherosclerosis plaques might be essential for generating osteochondrocyte-like cells that facilitate the development of extensive mineralized surfaces, contributing to plaque stabilization.

A recent histological study in human carotids proposed that TGF-β expressed by M2 macrophages could link inflammation resolution to osteochondrocyte-like cell differentiation and the growth of macrocalcifications [274]. Thus, M1 macrophages might be implicated in the detrimental cycle connecting microcalcification to inflammation, while M2 macrophages could aid in breaking this cycle and promoting plaque repair through ossification. The spatial distribution of macrophages in atherosclerotic plaques supports this notion, with proinflammatory M1 cells located in unstable, rupture-prone areas, while anti-inflammatory M2 macrophages are predominantly found in the vascular adventitia, farther from the lipid core and in more stable plaque regions [29].

M1 macrophages exhibit increased lipid accumulation, mitochondrial dysfunction, destabilized lysosomes, activated oxidative stress, defective efferocytosis, and elevated release of proinflammatory mediators, including IL-6, IL-12, IL-1β, and TNF-α, as well as reactive oxygen species. In contrast, M2 cells contain small lipid droplets and are thought to foster atherosclerosis regression by supplementing anti-inflammatory factors (IL-10 and TGF-β), facilitating efferocytosis, and promoting tissue repair through collagen formation and angiogenesis [275]. The balance between M1 and M2 macrophages likely plays a pivotal role in transitioning from proinflammatory plaques with microcalcifications to inflammation resolution associated with plaque macrocalcification. Interestingly, a comparable role for M1 and M2 macrophages is also strongly suspected during bone fracture healing [276].

## 11. Carotid Stenosis

Traditional methods for identifying atherosclerosis in the carotid artery focus on measuring the narrowing of the artery, identified as luminal stenosis. Severe stenosis is a key factor in defining a vulnerable plaque. Plaques with significant stenosis are at a higher risk of developing thrombosis and sudden blockages due to the influence of shear stress. Additionally, the presence of a stenotic plaque may signal the existence of other non-stenotic or less severely narrowed plaques that could be prone to rupture and thrombosis [8]. Recent research has explored molecular markers associated with the vulnerability of atherosclerotic plaques, particularly those related to severe stenosis. Oxidative stress and the inflammatory response are identified as major pathways in the development of atherosclerotic stenosis. In an interesting study [277], the authors demonstrated an elevation of sICAM-1 concentration that was independently associated with atherosclerosis of ICA origin and predominantly increased in patients with low-grade lesions and with clinical manifestations of vascular disorders. For instance, studies by Li X and colleagues demonstrated elevated circulating levels of various markers, including total oxidant status, lipid hydroperoxide, 8-isoprostane, malondialdehyde, monocyte chemotactic protein-4, amyloid A, hs-CRP, and TNF-α in elderly patients with both severe stenosis in the right carotid artery and severe multivessel coronary artery stenosis [130]. Furthermore, other studies found significantly increased circulating levels of malondialdehyde, oxidized LDL, homocysteine, F2-isoprostanes, TNF-α, hs-CRP, prostaglandin E2, and IFN-γ in patients with combined severe stenosis in both the carotid and coronary arteries [278]. In patients with carotid stenosis, the levels of TSG-6 (Tumor Necrosis Factor-Stimulated Gene-6) in the serum were higher. The TSG-6 expression increased in tissues with moderate and severe stenosis. Patients with symptomatic stenosis had higher serum TSG-6 levels compared to those with asymptomatic stenosis. TSG-6 could potentially be a new, easily measurable biomarker for the non-invasive screening of severe and symptomatic carotid stenosis in clinical practice [279]. Moreover, patients with symptomatic carotid stenosis showed significantly higher levels of NET markers than asymptomatic patients and healthy individuals. Increased levels of neutrophil–platelet aggregates led to the generation of NETs in symptomatic carotid stenosis. NETs contributed to platelet-derived microparticle (PCA) formation through TF in patients with carotid stenosis. NETs could be a potential biomarker and their inhibition could be a therapeutic target for preventing recurring strokes in severe carotid stenosis [280]. Damage to the endothelium is a crucial aspect of atherosclerosis. Researchers have hypothesized that increased levels of vWF, thrombomodulin, intercellular adhesion molecule-1, and E-selectin might be related to disease severity in patients with peripheral or carotid atherosclerosis. vWF was identified as the most sensitive marker for peripheral atherosclerosis. However, none of the plasma markers seemed to be useful for indicating the degree of carotid artery stenosis [6]. In individuals with high-grade internal carotid artery (ICA) stenosis, those with recent symptoms showed a higher level of plasma sCD36 compared to those with former symptoms [131]. Furthermore, serum macrophage CXC-chemokine ligand 16 (CXCL16) has been identified as a potential biomarker for carotid-vulnerable plaques.

The serum CXCL16 levels increase with plaque area, lumen stenosis rate, and intima–media thickness [132]. Patients with stenosis of 70% or more have higher levels of MIP-1α compared to those with stenosis below 70%. Significantly higher levels of CD14 are observed in patients with hypoechoic (vulnerable) lesions compared to those with hyperechoic (stable) lesions [133]. The platelet-to-lymphocyte ratio has been proposed as a novel indirect marker of inflammation. Yayla C et al. demonstrated that the platelet-to-lymphocyte ratio significantly increases in parallel with the severity of atherosclerosis [281]. In a compelling investigation [282], the researchers examined alterations in the concentrations of vascular endothelial growth factor (VEGF) and VEGF Receptor-2 (VEGFR-2) in individuals undergoing carotid endarterectomy. The study encompassed 43 patients with extracranial carotid stenosis exceeding 70%. The exclusion criteria included severe vertebrobasilar stenosis, recent (<1 month) vascular events (such as stroke, coronary infarction, arterial thromboembolism), critical lower extremity ischemia, recent infection, autoimmune disease, or malignancy. Blood samples were collected before CEA and on the second postoperative day, while a control group comprising thirty healthy blood donors was utilized for comparison. The researchers employed an enzyme-linked immuno-absorbent assay to determine the VEGF and VEGFR-2 levels.

The pre-operative levels of VEGF and VEGFR-2 were significantly elevated. Following CEA, there was a noteworthy reduction in both VEGF and VEGFR-2, although they did not return to normal values. Notably, in asymptomatic patients and those with contralateral carotid stenosis exceeding 50%, the decrease in VEGF levels did not reach statistical significance. Conversely, within the same subgroups, a substantial reduction in VEGFR-2 values was observed. The study highlighted a substantial increase in the serum levels of VEGF and VEGFR-2 in patients with severe carotid stenosis. These elevated pre-operative levels significantly decreased after endarterectomy, underscoring the pivotal role of these molecules in the progression of carotid disease. Nuotio et al. [134], in contrast to earlier studies, found that symptomatic carotid disease was not associated with increased expression of adhesion molecules in the endothelium of advanced carotid plaques or circulation but demonstrated reduced expression of adhesion molecules in the intima–media of symptomatic carotid plaques. Another research study [283] postulated that environments characterized by severe carotid atherosclerotic disease exacerbate endothelial dysfunction, thereby contributing to an elevated risk of recurrent cerebrovascular events. The researchers utilized nonischemic common carotid arteries from mice, mounted them in tissue baths for isometric contraction force measurements, and exposed them to serum obtained from men with a recent ischemic stroke exhibiting varying degrees of carotid stenosis: low- or moderate-grade stenosis (LMGS; <70%) and high-grade stenosis (HGS; ≥70%). The outcomes revealed that serum from stroke patients induced impairment of acetylcholine relaxations in the carotid arteries of mice, indicative of endothelial dysfunction. This effect was more pronounced when the carotid arteries were incubated with serum from patients experiencing a recurrent stroke or vascular death within 1 year of follow-up. Stratifying patients based on the degree of stenosis demonstrated that serum from HGS patients induced more prominent endothelial dysfunction in the carotid artery, a phenomenon associated with elevated circulating levels of IL-1β.

## 12. Conclusions

The specific mechanisms underlying plaque vulnerability and rupture remain elusive, encompassing various factors such as cap thinning, the expansion of the lipid core, the presence of microcalcifications, neoangiogenesis, elevated levels of inflammatory cytokines, and proteases that aid in matrix digestion. All cell types involved in atherosclerotic plaque formation play a role in plaque rupture and subsequent thrombosis. Inflammatory cells within the plaque, which convey molecular signals, are crucial for understanding the pathways leading to vulnerability. For instance, lymphocytes can release factors like CD-40L and macrophage-derived foam cells secrete cytokines, growth factors, tissue factor, IFN-γ, and MMPs, and generate reactive oxygen species. The accumulation of free cholesterol within the plaque strongly induces apoptosis of macrophage-derived foam cells and promotes the enlargement of the necrotic core. Even today, one of the errors in the approach to the study of carotid atherosclerotic pathology remains, perhaps, the consideration of plaque vulnerability and rupture as a one-dimensional process. Talking about a vulnerable patient rather than a vulnerable plaque helps to broaden the scope of observation and allows for a well-rounded study that also takes into consideration the molecular mechanisms underlying the risk associated with potential plaque rupture. Current evidence demonstrates that plaque vulnerability phenotypes are multiple and heterogeneous and are associated with many highly complex molecular pathways that determine the activation of an immune-mediated cascade that culminates in thromboinflammation. We do not know if there are as many types of vulnerable patients whose exhibition of specific signals and consequent activation of specific pathways induce the manifestation of plaque vulnerability towards one phenotype rather than another. In the future, research will have to focus on the individual molecular triggers underlying the expression of individual plaque vulnerability which may represent diagnostic markers and potential therapeutic targets for more personalized medicine.

## Figures and Tables

**Table 1:** Principle molecular patterns associated with specific vulnerability characteristics.

Features of Plaque Vulnerability	Molecular Patterns Associated with Plaque Instability	References
**Plaque Rupture And Atherothrombosis**	GPIba, vWF, TF, thrombin, CRP, IL-1, NETs	[70,72,73,74,75,76,78]
**Plaque Ulceration**	MMP-9, CD40, CD40L, heat shock protein 27, thrombospondin-1, ApoE, vWF, ADAMTS13	[37,86,87,88,89,90]
**Fibrous Cap Thickness**	TF, IFN-γ, MMPs, reactive oxygen species, IL-18, growth differentiation factor-15, TNF, PDGF B, macrophage-produced heparin-binding epidermal growth-factor-like growth factor, cadherin, prostaglandins, ACE/Ang II, XCR1, CD177	[91,92,93,94,95,96,97,98,99]
**Intraplaque Hemorrhage**	VEGF, MMPS, bFGF, vWF, VE-cadherin, E-selectin, intercellular adhesion molecule-1, vascular cell adhesion molecule-1, Semaphorin 7A	[100,101,102,103,104,105,106,107,108]
**Large Lipidic Core**	CD36, ABCA, LP-PLA2, LPA, 7β-OH-cholesterol, C3d, C5b-9 neoantigen, ACAT	[109,110,111,112,113,114,115,116,117]
**Microcalcification**	TNF-α, IL-1β, IL-6, BMP2, IL-8, OPN, OPG, TGF-β	[118,119,120,121,122,123,124,125,126,127,128,129,130]
**Carotid Stenosis**	Lipid hydroperoxide, 8-isoprostane, malondialdehyde, monocyte chemotactic protein-4, amyloid A, hs-CRP, TNF-α, prostaglandin E2, IFN-γ, TSG-6, vWF, CXCL16, VEGF, VEGFR2	[5,131,132,133,134]

ABCA: ATP binding cassette A; ACAT: acylcoenzyme A–cholesterol acyltransferase; ACE/Ang II: angiotensin-converting enzyme/angiotensin II; ADAMTS: A Disintegrin and metalloproteinase with Thrombospondin motifs; ApoE: apolipoprotein E; bFGF: basic fibroblast growth factor; BMP2: bone morphogenetic protein 2; CXCL: eC-X-C motif chemokine ligand; CRP: C-reactive protein; hs-CRP: high-sensitivity C-reactive protein; IFN-γ: interferon-gamma; IL: interleukin; LPA: Lysophosphatidic acid; MMP: matrix metalloprotease; NETs: neutrophil extracellular traps; OPN: osteopontin; OPG: osteoprotegerin; GP: platelet glycoprotein; PDGF: platelet-derived growth factor; TF: tissue factor; TGF: transforming growth factor; TSG-6: Tumor Necrosis Factor-Stimulated Gene-6; TNF: tumor necrosis factor; VEGF: vascular endothelial growth factor; vWF: von Willebrand factor.

## Data Availability

Not applicable.

## Abbreviations

ABC: ATP binding cassette; ACAT: acylcoenzyme A–cholesterol acyltransferase; ACE/Ang II: angiotensin-converting enzyme/angiotensin II; ADAMTS: A Disintegrin and metalloproteinase with Thrombospondin motifs; ApoE: apolipoprotein E; bFGF: basic fibroblast growth factor; BMP2: bone morphogenetic protein 2; CXCL: eC-X-C motif chemokine ligand; CRP: C-reactive protein; ECs: endothelial cells; ECM: extracellular matrix; FGF: fibroblast growth factor; HDL: high-density lipoprotein; hs-CRP: high-sensitivity C-reactive protein; IFN-γ: interferon-gamma; IL: interleukin; IPH: intraplaque hemorrhage; LAAS: large-artery atherosclerosis; LDL: low-density lipoprotein; LNRC: lipid-rich necrotic core; Lp-PLA2: lipoprotein-associated phospholipase A2; LPA: Lysophosphatidic acid; MGP: matrix Gla protein; MES: microembolic signals; MMP: matrix metalloprotease; NETs: neutrophil extracellular traps; NLRP3: NOD-like receptor family pyrin domain containing 3; OPN: osteopontin; OPG: osteoprotegerin; OX-LDL: oxidized low-density lipoprotein; GP: platelet glycoprotein; PDGF: platelet-derived growth factor; PMPs: platelet-derived microparticles; RANKL: receptor activator of NF-κB ligand; RBCs: red blood cells; TCD: transcranial Doppler; TF: tissue factor; TGF: transforming growth factor; TOAST: trial of ORG 10,172 in the acute stroke treatment; TSG-6: Tumor Necrosis Factor-Stimulated Gene-6; TNF: tumor necrosis factor; VEGF: vascular endothelial growth factor; VSCM: vascular smooth muscle cell; vWF: von Willebrand factor.
